# A Novel Missense Variant in SORBS2 Is Causative With Familial Alzheimer's Disease

**DOI:** 10.1111/cns.70256

**Published:** 2025-02-06

**Authors:** Qi Wang, Shiyuan Wang, Shuman Cao, Qigeng Wang, Yiping Wei, Ying Li, Yan Wang, Yan Li, Wei Qin, Meina Quan, Jianping Jia

**Affiliations:** ^1^ Innovation Center for Neurological Disorders and Department of Neurology, Xuanwu Hospital Capital Medical University, National Clinical Research Center for Geriatric Diseases Beijing People's Republic of China; ^2^ Beijing Key Laboratory of Geriatric Cognitive Disorders Beijing People's Republic of China; ^3^ Clinical Center for Neurodegenerative Disease and Memory Impairment Capital Medical University Beijing People's Republic of China; ^4^ Center of Alzheimer's Disease, Beijing Institute of Brain Disorders, Collaborative Innovation Center for Brain Disorders Capital Medical University Beijing People's Republic of China; ^5^ Key Laboratory of Neurodegenerative Diseases Ministry of Education Beijing People's Republic of China

**Keywords:** Alzheimer's disease, neuroinflammation, pathological mechanism, *SORBS2*

## Abstract

**Background:**

Alzheimer's disease (AD) is a common neurodegenerative disorder with a substantial genetic component. Despite advances in elucidating the genetic underpinnings of AD, much of its heritability remains unexplained. Discovering novel genetic variants and understanding their pathogenic roles are crucial challenges in AD research.

**Objective:**

This study aimed to identify pathogenic genes and elucidate their role in familial early‐onset AD (EOAD).

**Methods:**

Blood samples from an EOAD pedigree and Sorbin and SH3 Domain‐Containing Protein 2 (*SORBS2*) T189M transgenic mice were analyzed. Cognitive function was assessed via the Morris water maze (MWM). Protein expression was evaluated by western blotting, while amyloid‐β (Aβ) levels were quantified via immunohistochemistry and enzyme‐linked immunosorbent assay. Inflammatory markers were measured using immunofluorescence and quantitative reverse transcription polymerase chain reaction (PCR). Neuronal morphology, including dendritic and spine alterations, was examined using Golgi staining.

**Results:**

We identified a novel *SORBS2* variant (c. 566C>T, p. T189M) in a Han Chinese family, segregating with AD in a Mendelian fashion. *SORBS2* T189M transgenic mice exhibited cognitive deficits, cortical Aβ accumulation, and an increased Aβ42/Aβ40 ratio. Additionally, elevated levels of interleukin (IL)‐1β, IL‐6, tumor necrosis factor α (TNF‐α), and ionized calcium‐binding adaptor molecule 1 (Iba1)‐positive microglia, along with neuronal loss, were observed in the brains of T189M mice.

**Conclusion:**

Our study suggest that the *SORBS2* T189M variant is a novel candidate causal mutation associated with familial AD in a Chinese pedigree, contributing to AD pathogenesis by promoting neuroinflammation and neuronal injury. Notably, this study is the first to establish a link between *SORBS2* mutations and AD.

## Graphical abstract

A SORBS2 gene variant (c.566C>T, p.T189M) was identified in an EOAD family. Overexpression of SORBS2 T189M variant in a murine model activated microglia, up‐regulated inflammatory cytokines, induced neuroinflammation, exacerbated Aβ accumulation, accelerated neurodegeneration, and ultimately led to cognitive impairment. These results suggest that SORBS2 T189M variant is pathogenic in AD.

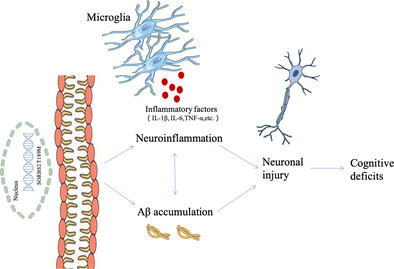

## 1 Introduction

Alzheimer's disease (AD) is the most prevalent neurodegenerative disorder, characterized by a substantial genetic predisposition [1]. Although numerous common genetic variants have been implicated in AD through genome‐wide association studies, their contributions to disease susceptibility are relatively minor, with the exception of the *APOE* ε4 alleles [2]. Advances in next‐generation sequencing have identified rare variants in several loci that confer moderate to high risk compared to more common variants [3, 4]. The most well‐characterized pathogenic variants in AD are fully penetrant mutations in the amyloid‐β precursor protein (*APP*) and presenilin (*PSEN*) 1 and 2 genes [5]. These mutations perturb amyloid‐β (Aβ) peptide processing, implicating Aβ aggregation as a critical early event in AD pathogenesis [6]. *APP* and *PSEN1/2* mutations, which are found in families with dominantly inherited, early‐onset AD (onset before 65 years of age), account for approximately 10% of early‐onset cases [7, 8]. Other rare variants, primarily identified in genes such as sortilin‐related receptor [9], triggering receptor expressed on myeloid cells 2 [10] and ATP‐binding cassette subfamily A member 7 [11], have been identified through whole‐exome and whole‐genome sequencing. Recently, our research group identified a novel candidate pathogenic mutation, *ZDHHC21* p.T209S, in a Chinese familial Alzheimer's disease (FAD) pedigree [12]. This mutation likely represents a novel pathogenic mechanism in AD by disrupting protein palmitoylation [12], underscoring the polygenic architecture of AD, with many causative and risk alleles yet to be elucidated. The incomplete understanding of the mechanistic impact of genetic variants remains a significant barrier to identifying novel therapeutic targets for AD, highlighting the importance of resolving the “missing heritability” in AD research [6]. This study aimed to identify pathogenic genes and elucidate their role in familial early‐onset AD (EOAD).

In this study, we identified a *SORBS2* gene variant (c.566C>T, p.T189M) in a Han Chinese family with EOAD following an autosomal‐dominant inheritance pattern. Our findings demonstrate that overexpression of the *SORBS2* T189M variant in a murine model induces neuroinflammation, exacerbates Aβ pathology, accelerates neurodegeneration, and culminates in cognitive impairment. These results suggest that the *SORBS2* T189M variant is pathogenic in AD, providing novel insights into the role of *SORBS2* variants in AD pathogenesis.

## 2 Materials and Methods

### 2.1 Human Subjects

**FIGURE 1:**
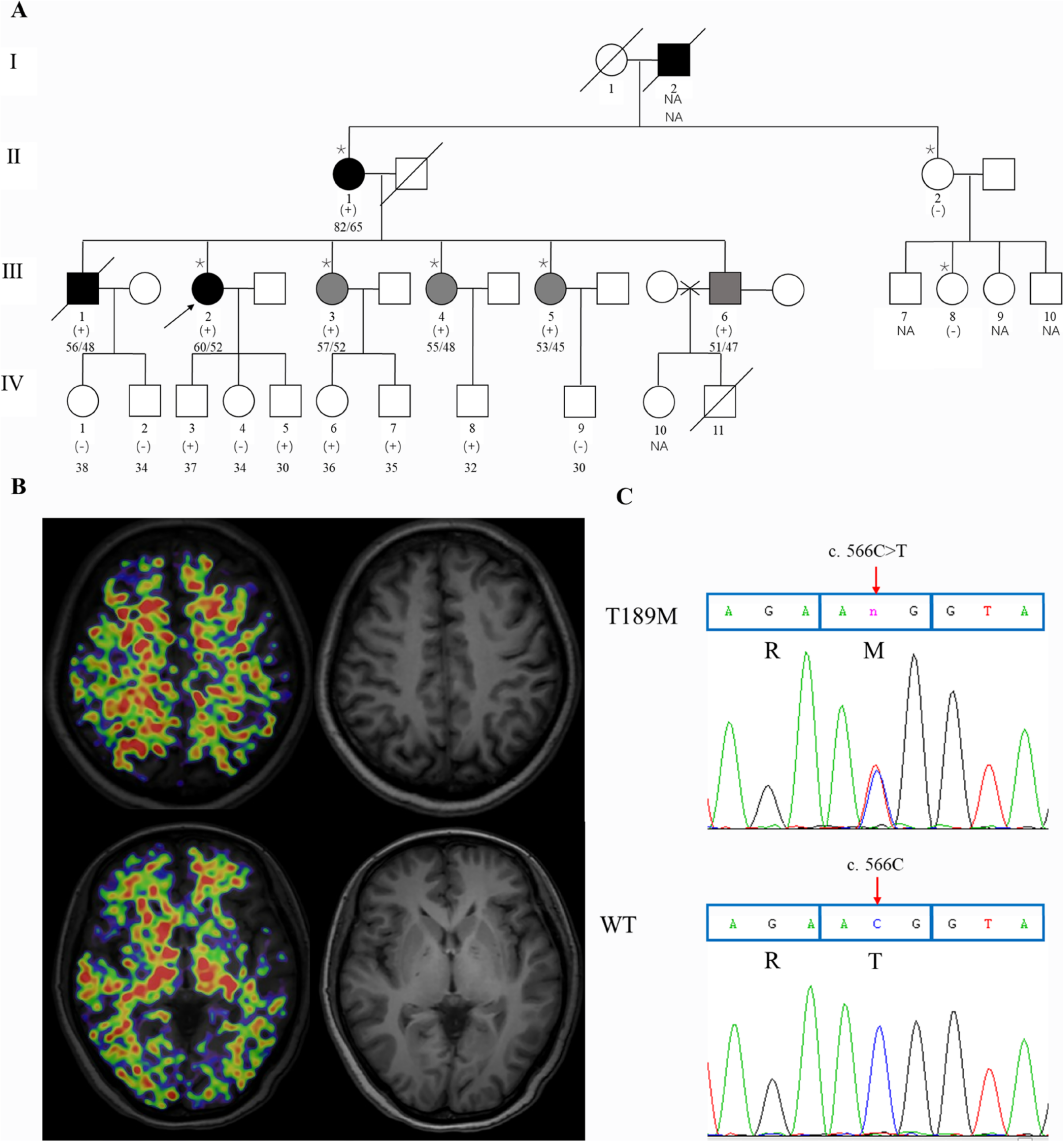
Identification of *SORBS2* T189M variant in a Han Chinese family with early‐onset Alzheimer's disease (EOAD). (A) A multigenerational pedigree showing individuals affected by EOAD. Affected members (indicated by 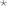) underwent whole genome sequencing. The proband is indicated by an arrow; black symbols represent affected members, white symbols represent unaffected members, squares indicate males, circles indicate females, and slashes denote deceased members. Age1/age2 in the top left corner of symbols indicates current age or age at death/age at onset. (+) indicates mutation carriers, and (−) indicates non‐carriers. NA indicates data not available. (B)^11^C‐PIB PET imaging of the proband confirms amyloid deposition, consistent with AD pathology. (C) Sequencing chromatogram showing the heterozygous *SORBS2* c.566C>T mutation in affected family members.

Participants were recruited from the Chinese Familial Alzheimer's Disease Network (CFAN) (https://www.clinicaltrials.gov/; NCT03657732). Diagnoses of AD were based on criteria established by the National Institute on Aging and Alzheimer's Association [13], while amnestic mild cognitive impairment (aMCI) was diagnosed according to previously validated protocols [14]. The family (Figure 1A) consisted of 23 members, including 8 individuals with AD or aMCI (age at onset [AAO] ranging from 45 to 65 years), 11 unaffected individuals, and 4 individuals with uncertain EOAD status. Samples were collected in accordance with the principles of the Declaration of Helsinki, and the informed consents were obtained from all the participants or their legal representatives supervisors of the patients. This study was approved by the Ethics Committee of Xuanwu Hospital, Capital Medical University (Approval No.: LYS [2019] 110).

### 2.2 Whole‐Genome Sequencing (WGS) of the AD Pedigree

Following the exclusion of *PSEN1/2* and *APP* gene mutations via Sanger sequencing, WGS was conducted on 7 family members (5 patients and 2 cognitively normal individuals). High‐quality genomic DNA samples were sheared into 350 bp fragments using Covaris sonication. Paired‐end DNA libraries were constructed using the Illumina TruSeq Library Construction Kit according to the manufacturer's protocols. Sequencing was performed on the Illumina NovaSeq platform (150 bp paired‐end reads, > 30 × coverage). Raw reads were filtered and aligned to the UCSC hg19 human reference genome using BWA (Version: 0.7.15‐r1140). Variant calling was conducted using GATK (Version: 4.1.2.0–1) to detect single nucleotide polymorphisms (SNPs), insertions, and deletions. Potentially pathogenic variants were identified based on criteria detailed in Figure S1 and analyzed using 13 in silico prediction algorithms. A mutation was considered potentially deleterious if it met thresholds for SIFT < 0.05 [15], PolyPhen2 > 0.8 [16], CADD > 20, CADD raw > 2 [17], GERP++RS > 4 [18], M‐CAP > 0.025 [19], LoFtool < 0.5 [20], and predictions from Condel [21], DANN [22], FATHMM [23], LRT [24], MetaLR [25], MutationTaster [26], and ROVEAN [27]. A cumulative damaging variant score (range: 0–20) was calculated to prioritize the most likely pathogenic variants, with higher scores indicating greater pathogenic potential. All candidate pathogenic variants were validated by Sanger sequencing at Beijing Ruibio BioTech Co. Ltd., and co‐segregation analysis was performed as previously described [28].

### 2.3 Generation of the *SORBS2* T189M Mouse Model

All animal models were obtained from the Shanghai Model Organisms Center. Transgenic mice overexpressing human *SORBS2* wild‐type (hSORBS2) or *SORBS2* T189M mutations (T189M) were generated using random transgenesis. Constructs containing the MoPrP promoter and the *SORBS2* coding region, with or without the mutation, were microinjected into C57BL/6 zygotes. Transgenic mice were then backcrossed to generate colonies. All experimental protocols were approved by the Ethics Committee of Capital Medical University (Approval No. AEEI‐2017‐004).

Male mice with hSORBS2 or T189M, aged 3, 6, and 9 months, were housed under controlled conditions (22°C–24°C, 40%–60% humidity, 12‐h light/dark cycle), with ad libitum access to food and water. Genotypes were confirmed by polymerase chain reaction (PCR). Transgenic mice (9 months old) were used as the experimental model, with age‐matched wild‐type (WT) littermates serving as control.

### 2.4 Enzyme‐Linked Immunosorbent Assay (ELISA)

Cortical tissues were homogenized in phosphate‐buffered saline (PBS) containing a protease inhibitor cocktail, briefly sonicated, and centrifuged. Supernatants were analyzed for soluble Aβ40 and Aβ42 levels using ELISA kits (CSB‐E08300m and CSB‐E10787m, CUSABIO), according to the manufacturer's instructions. Data were normalized to total protein concentration.

### 2.5 Quantitative Reverse Transcription Polymerase Chain Reaction (qRT‐PCR)

Total RNA was isolated using the Tissue Total RNA Kit (Yeasen) and reverse transcribed into complementary DNA using a commercial kit (Yeasen). Complementary DNA was amplified with Hieff UNICON Universal Blue qPCR SYBR Green Master Mix (Yeasen). The qRT‐PCR was performed on a StepOne Plus Real‐time PCR System (Applied Biosystems) under the following conditions: pre‐incubation at 95°C for 2 min, followed by 40 cycles of 95°C for 10 s (denaturation) and 60°C for 30 s (annealing). Primer sequences were provided in Table S1. Relative mRNA expression levels were normalized to glyceraldehyde 3‐phosphate dehydrogenase (*GAPDH*) and calculated using the 2^−△△Ct^ method.

### 2.6 Western Blotting

The right cerebral hemisphere from each murine specimen was homogenized in radio immunoprecipitation assay (RIPA) buffer (Applygen), supplemented with protease and phosphatase inhibitor cocktails, followed by sonication and centrifugation at 10,000 × *g* for 30 min. The resultant supernatants were collected, and protein concentrations were quantified using a BCA assay (Beyotime). Proteins were denatured at 99°C for 10 min in the loading buffer. Subsequently, proteins were resolved via sodium dodecyl sulfate‐polyacrylamide gel electrophoresis (SDS‐PAGE) and transferred onto polyvinylidene (PVDF) membranes (Millipore). Membranes were blocked with 5% non‐fat dry milk in Tris‐buffered saline with 0.1% Tween 20 (TBS‐T) for 1 h at room temperature, followed by overnight incubation at 4°C with primary antibodies: anti‐SORBS2 (Proteintech 24643‐1‐AP, 1:1000) and anti‐GAPDH (Proteintech 60004‐1‐IG, 1:2000). Following this, they were incubated with horseradish peroxidase (HRP)‐conjugated secondary antibodies for 2 h at room temperature and visualized using enhanced chemiluminescence (ECL) detection. Densitometric analysis of protein bands was performed using ImageJ software.

### 2.7 Morris Water Maze Assay

Cognitive function, specifically spatial learning and memory, in 9‐month‐old T189M transgenic mice was assessed using the Morris water maze (MWM). The apparatus consisted of a circular pool (120 cm diameter, 50 cm depth) with a submerged platform (10 cm diameter). Water temperature was maintained at 22°C ± 1°C. The test protocol spanned six consecutive days consisting of a visible platform stage (day 1), a hidden platform stage (days 2–5), and a probe trial stage (day 6). On day 1, a visible cue was affixed to the platform. During the hidden platform stage, the cue was removed, and the platform was submerged 1 cm beneath the water surface. Mice underwent four training trials per day during the first two stages, and the time to locate the platform (escape latency) was recorded. If a mouse did not find the platform within 60 s, the escape latency was recorded as 60 s, following which the mouse was placed on the platform for 20 s. On day 6, the probe trial was conducted by removing the platform and allowing the mice to swim for 60 s. Time spent in the target quadrant and platform crossings were recorded using the SMART 3.0 tracking system.

### 2.8 Immunohistochemistry

Following transcardial perfusion with PBS (Solarbio), the left cerebral hemisphere was post‐fixed in 4% paraformaldehyde for 24 h. The tissue was subjected to dehydration via a graded ethanol series, clear in xylene, embedded in paraffin, and coronally sectioned (5 μm thickness). Sections were deparaffinized in xylene and rehydrated through graded ethanol concentrations. Antigen retrieval was performed using citrate buffer, followed by incubation with 0.3% hydrogen peroxide (H_2_O_2_) to quench endogenous peroxidase activity. Sections were blocked with 5% normal goat serum at 37°C for 1 h, then incubated overnight at 4°C with a primary antibody against Aβ (Abcam ab201060, 1:1000). After washing, sections were incubated with a secondary antibody goat anti‐rabbit IgG H&L (Abcam ab6721, 1:1000) in PBS for 2 h at 37°C. Visualization was achieved using a DAB substrate kit (Solarbio), and slides were examined under a scanning microscope (Leica Microsystems Inc).

### 2.9 Immunofluorescence Staining

Paraffin‐embedded brain sections (4 μm thickness) were processed for immunofluorescence staining. Sections were deparaffinized in xylene, rehydrated through a graded ethanol series, and subjected to antigen retrieval using either sodium citrate (pH 6.0) or Tris–EDTA (pH 9.0). Following blocking, sections were incubated overnight at 4°C with primary antibodies against IL‐1β (Abcam ab254360, 1:100), IL‐6 (Abcam ab208113, 1:100), TNF‐α (Abcam ab1793, 1:50), and anti‐Iba1 (Abcam ab178846, 1:100). Following primary incubation, sections were incubated with a fluorophore‐conjugated secondary antibody goat anti‐rabbit IgG H&L (Abcam ab150080, 1:1000) for 2 h at 37°C. Nuclei were counterstained with DAPI (Solarbio), and slides were mounted with an anti‐fade medium. Imaging was performed using a confocal laser scanning microscope (Zeiss).

### 2.10 Golgi‐Cox Staining

Neurons within the external granular layer of the left cerebral hemisphere were visualized using the FD Rapid GolgiStain Kit (FD Neuro Technologies Inc.). Briefly, frozen brain sections (100 μm thickness) were cut coronally, dried at room temperature in the dark, rinsed with distilled water, and stained with a developing solution. Sections were dehydrated through a graded ethanol series (50%, 75%, 95%, and 100%), cleared in xylene, mounted with resin (Yeasen), and examined under a microscope (Nikon). Neuronal morphology, including total dendritic length, branching points, and spine density, was analyzed using Sholl analysis with the FIJI image processing package, adhering to established protocols [12, 29].

### 2.11 Statistical Analysis

Data were presented as mean ± standard error of the mean (SEM). All data were subjected to test for normality using Shapiro–Wilk test or Kolmogorov–Smirnov test. One‐way analysis of variance (ANOVA) followed by Tukey's multiple comparisons test(homogeneity of variance) or Dunnett's T3 multiple comparisons test(non‐homogeneity of variance) were used to detect the difference among the three groups for normally distributed data. Data that did not exhibit a normal distribution were analyzed using a nonparametric equivalent (Kruskal–Wallis test). Two‐way ANOVA followed by Dunnett's multiple comparisons test and Tukey's multiple comparisons test were utilized for escape latencies in the MWM test and the number of dendritic branches from the soma. GraphPad Prism version 9.0 (GraphPad Prism Software, La Jolla, CA, USA) was utilized for all statistical analyses. The *p*‐value < 0.05 was considered statistically significant.

## 3 Results

### 3.1 Proband and Family Members

The proband (III‐2) presented to our neurology department in 2016 with a history of progressive memory decline that commenced 8 years prior, with significant exacerbation in the past year. Clinical manifestations included episodic memory lapses, misplacing objects, repetitive questioning, failure to turn off cooking appliances, and confusion regarding the names of acquaintances. Neuropsychological assessment revealed marked cognitive impairment, with a Mini‐Mental State Examination (MMSE) score of 24 and a Montreal Cognitive Assessment (MoCA) score of 24, both below the normative thresholds. The patient's Activities of Daily Living (ADL) score was 20, and her Clinical Dementia Rating (CDR) global score was 1, indicating a substantial impact on her functional abilities. Executive function, attention, praxis, language comprehension, and visuospatial orientation were notably impaired. Additional symptoms included affective liability, feelings of guilt, and insomnia. Mild bilateral hippocampal atrophy was detected on magnetic resonance imaging (MRI). In addition, ^11^C‐labeled Pittsburgh Compound‐B positron emission tomography (^11^C‐PIB PET) imaging demonstrated significant Aβ deposition in the bilateral frontal, parietal, occipital, and lateral temporal cortices, with the most pronounced accumulation in the right temporo‐occipital lobe (Figure 1B).

In addition to the proband, five other members of this FAD pedigree exhibited cognitive decline (Table 1). The proband's mother (II‐1) was diagnosed with probable AD at age 65, with an MMSE score of 5 and a MoCA score of 3, rendering her unable to complete additional cognitive assessments. By age 80, she was bedridden and fully dependent on others for daily activities. The proband's eldest brother (III‐1) began exhibiting short‐term memory impairment at age 48 and was subsequently diagnosed with possible AD. Her eldest sister (III‐3) developed short‐term memory decline at age 52, along with object misplacement, conversation forgetfulness, insomnia, and personality changes. Her second sister (III‐4) showed episodic memory deficits at age 58, followed by a decline in executive function, and eventually became indifferent to her family and grandchildren, with significant communication difficulties in recent years. Her youngest sister (III‐5) presented with memory loss at age 45, progressing to impaired executive function and eventually losing the ability to perform familiar tasks, such as sewing. Her youngest brother (III‐6) was diagnosed with aMCI at age 47, exhibiting similar clinical features to the proband, including forgetfulness and personality changes.

**TABLE 1:** Summary of WGS reports from early‐onset familial Alzheimer's disease pedigree.

Individual number	II‐1	III‐2	III‐3	III‐4	III‐5	II‐2	III‐8
Gene	*SORBS2*	*SORBS2*	*SORBS2*	*SORBS2*	*SORBS2*	*SORBS2*	*SORBS2*
Nucleotide change	c. 566C>T	c. 566C>T	c. 566C>T	c. 566C>T	c. 566C>T	No change	No change
Clinical diagnosis	AD	AD	aMCI	aMCI	aMCI	NC	NC
Years of education	0	7	0	8	8	0	5
CDR (0–3)	3	1	0.5	0.5	0.5	0	0
MMSE (0–30)	5	24	21	26	27	17	28
MoCA (0–30)	2	24	15	27	25	16	24
WHO‐UCLA Verbal Learning Test (0–45)	NA	19	26	29	24	NA	NA
WHO‐UCLA Delayed Recall genotype (0–15)	NA	7	0	13	10	NA	NA
APOE	ε4/ε3	ε3/ε3	ε3/ε3	ε4/ε3	ε4/ε3	ε3/ε3	ε3/ε3
^11^C‐PIB PET	NA	Aβ positive	NA	NA	NA	NA	NA
Clinical features	Memory impairment; impaired execution	Memory impairment; impaired execution; disorientation; insomnia; poor comprehension; personality changes	Memory impairment; personality changes	Memory impairment; personality changes	Memory impairment; personality changes	NA	NA

Abbreviations: NA, not available; NC, normal cognition.

### 3.2 Genetic Analysis and Variant Interpretation

WGS was performed on seven family members, including the proband (III‐2, female, 60 years) and her mother (II‐1, female, 82 years), both diagnosed with AD. Additionally, WGS was conducted on her eldest sister (III‐3, female, 57 years), second sister (III‐4, female, 55 years), and youngest sister (III‐5, female, 53 years) who had aMCI, as well as on her aunt (II‐2, female) and one cousin (III‐8, female), who had normal cognitive functions. No pathogenic mutations were identified in the common AD‐associated genes (*APP*, *PSEN1*, *PSEN2*) or other neurodegeneration‐related genes. The *APOE* genotypes of the family members were detailed in Table 1. One patient with AD (II‐1) and two patients with aMCI (III‐4, III‐5) were *APOE* ε4 carriers, while the proband (III‐2), one patient with aMCI (III‐3) and two cognitively intact family members (II‐2, III‐8) were non‐carriers of the *APOE* ε4 allele. The distribution of *APOE* ε4 alleles did not correspond with the familial pattern of cognitive decline.

Given the possibility of novel pathogenic mutations contributing to EOAD in this pedigree, rare and potentially deleterious variants co‐segregating with AD were investigated. Our findings showed that the proband harbored 7 potentially damaging variants, including *SORBS2* p.T189M, *WDR65/CFAP57* p.R1117Q, *ZFR* p.N367S, *ITGA2* p.R757C, *GARS* p.E738K, *PLA2G4F* p.R487C, and *UBR1* p.S827L. Thirteen in silico prediction tools were utilized to evaluate the potential pathogenicity of these variants. Among these candidates, the *SORBS2* variant (GenBank: NM_001270771.1: c.566C>T, rs374906705, p.T189M) yielded the highest pathogenicity score (17), while the other six variants scored below 12 (Table S2). The *SORBS2* T189M variant was present in a heterozygous state in the proband (Figure 1C), with a minor allele frequency (MAF) of 0.00003295 in the ExAC database, 0.00001589 in the gnomAD database, and 0.0001997 in the 1000 Genomes database. Multiple predictive models, including SIFT, PolyPhen‐2, fathmm‐MKL, fathmm‐XF, LRT, PROVEAN, and MutationTaster, along with comprehensive scoring algorithms like CADD, DANN, CONDEL, M‐CAP, and GERP++RS, consistently classified the *SORBS2* T189M variant as pathogenic. Sanger sequencing validated this variant, and its co‐segregation with cognitive disorders in heterozygous carriers was observed across all affected family members with AD or aMCI. However, further functional studies are required to establish the pathogenicity of the *SORBS2* T189M variant.

### 3.3 *SORBS2* T189M Mutation Induces Cognitive Impairment in Mice

**FIGURE 2:**
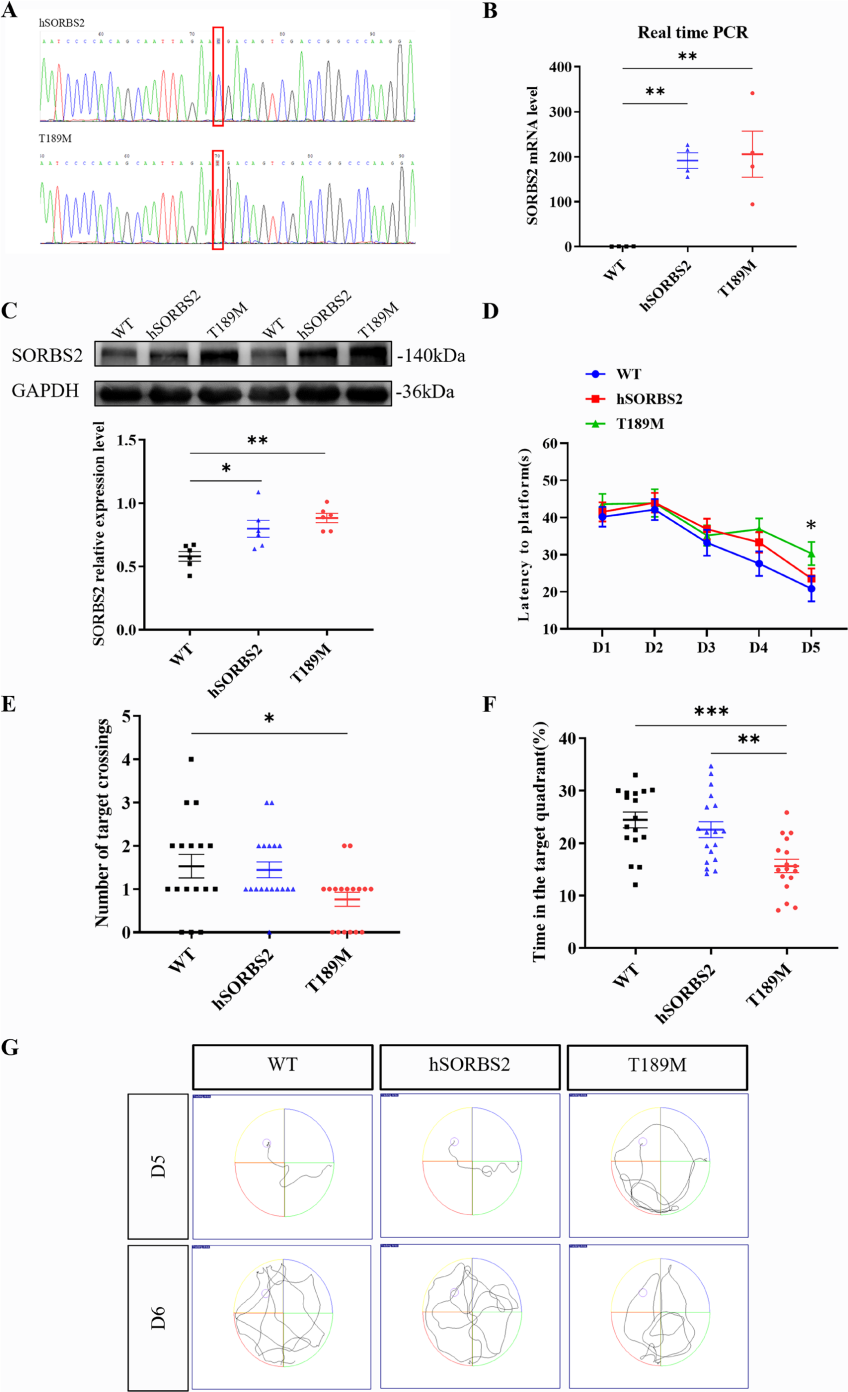
*SORBS2* T189M mutation decreased learning and memory abilities in transgenic mice. (A) Representative sequencing results for the *SORBS2* gene in transgenic mice. (B) Representative qRT‐PCR analysis showing *SORBS2* mRNA level in the brains of 6‐month‐old transgenic mice. (C) Representative western blotting analysis showing SORBS2 protein level in 6‐month‐old transgenic mice. (D–G) Morris water maze (MWM) test results showing latency to a platform (D), number of platform crosses (E), time spent in the target quadrant (F), and swim traces (G) in T189M mice. *n* = 4 or 6 mice/genotype; error bars SEM; one‐way ANOVA; *p < 0.05; **p < 0.01; ***p < 0.001 for (A–C); *n* = 17 mice/genotype; error bars SEM; two‐way ANOVA; *p < 0.05; **p < 0.01; ***p < 0.001 for (D–G). hSORBS2, Human *SORBS2* wild‐type overexpression; T189M, *SORBS2* T189M mutation; WT, wild type.

To investigate the pathogenic potential of the *SORBS2* T189M mutation, transgenic mice overexpressing the human wild‐type *SORBS2* (hSORBS2) and T189M mutant (T189M) genes were generated via random transgenic technology. Genotyping confirmed the presence of the *SORBS2* transgene, and sequencing revealed the introduction of the novel missense mutation [p.T189M] in these mice (Figure 2A). Subsequent analysis demonstrated increased RNA and protein levels of SORBS2 in the transgenic mice compared to WT (Figure 2B,C), confirming the successful establishment of the transgenic model. The impact of the *SORBS2* mutation on spatial learning and memory was evaluated using the MWM test (Figure 2D–G). While all groups, including WT, hSORBS2, and T189M mice, exhibited comparable swim speeds, the T189M mice showed a significantly prolonged latency to locate the platform on the fifth day of training compared to WT (Figure 2D). In the probe trial, T189M mice exhibited markedly reduced memory retention, as evidenced by fewer platform crossings, decreased time spent in the target quadrant, and altered swim paths compared to WT mice (Figure 2E–G). These results suggest that the *SORBS2* T189M mutation impairs spatial memory in mice.

### 3.4 *SORBS2* T189M Mutation Promotes Aβ Accumulation in Mice

**FIGURE 3:**
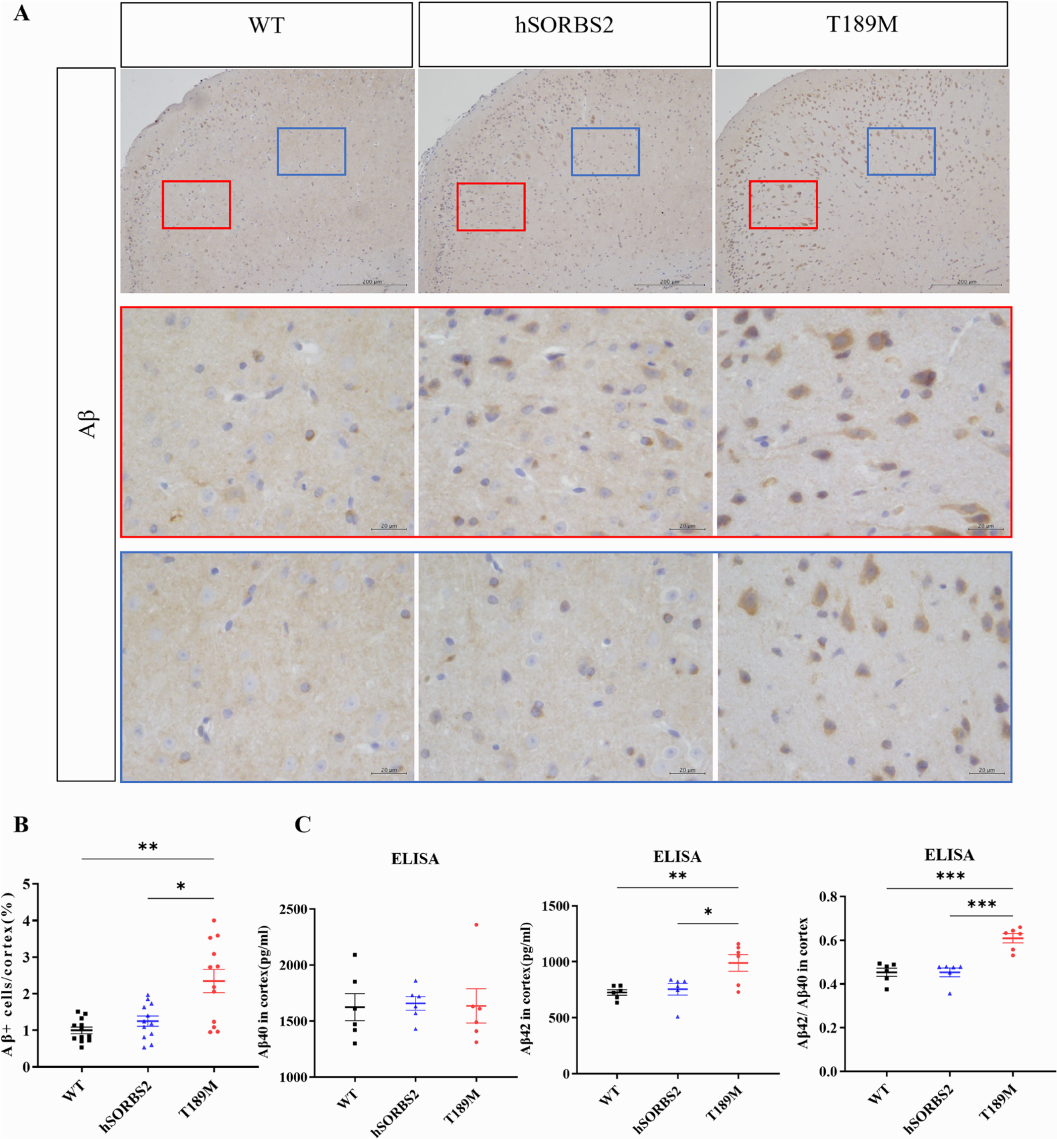
*SORBS2* T189M mutation increased Aβ in the cerebral cortex of transgenic mice. (A) Representative immunohistochemistry images of Aβ deposition in the cortex of 9‐month‐old WT, hSORBS2, and T189M mice. (B) Quantification of Aβ‐positive cells in the cortex. (C) ELISA results showing Aβ40, Aβ42, and Aβ42/Aβ40 levels in the cortex of 9‐month‐old WT, hSORBS2, and T189M mice. *n* = 4 or 6 mice/genotype; error bars SEM; 3 fields from 4 mice per group (B); one‐way ANOVA; *p < 0.05; **p < 0.01; ***p < 0.001. hSORBS2, Human SORBS2 wild‐type overexpression; T189M, SORBS2 T189M mutation; WT, wild type.

Given the familial origin of the *SORBS2* T189M mutation in a FAD pedigree, AD‐related neuropathological changes were assessed in the transgenic mice. Immunohistochemical analysis revealed increased intraneuronal Aβ deposition in the cortex of T189M mice (Figure 3A,B). Quantitative analysis demonstrated elevated levels of Aβ42 and an increased Aβ42/Aβ40 ratio in T189M mice compared to WT and hSORBS2 (Figure 3C). These findings indicate that the *SORBS2* T189M mutation facilitates Aβ accumulation, consistent with AD pathology.

### 3.5 *SORBS2* T189M Mutation Exacerbates Neuroinflammation

**FIGURE 4:**
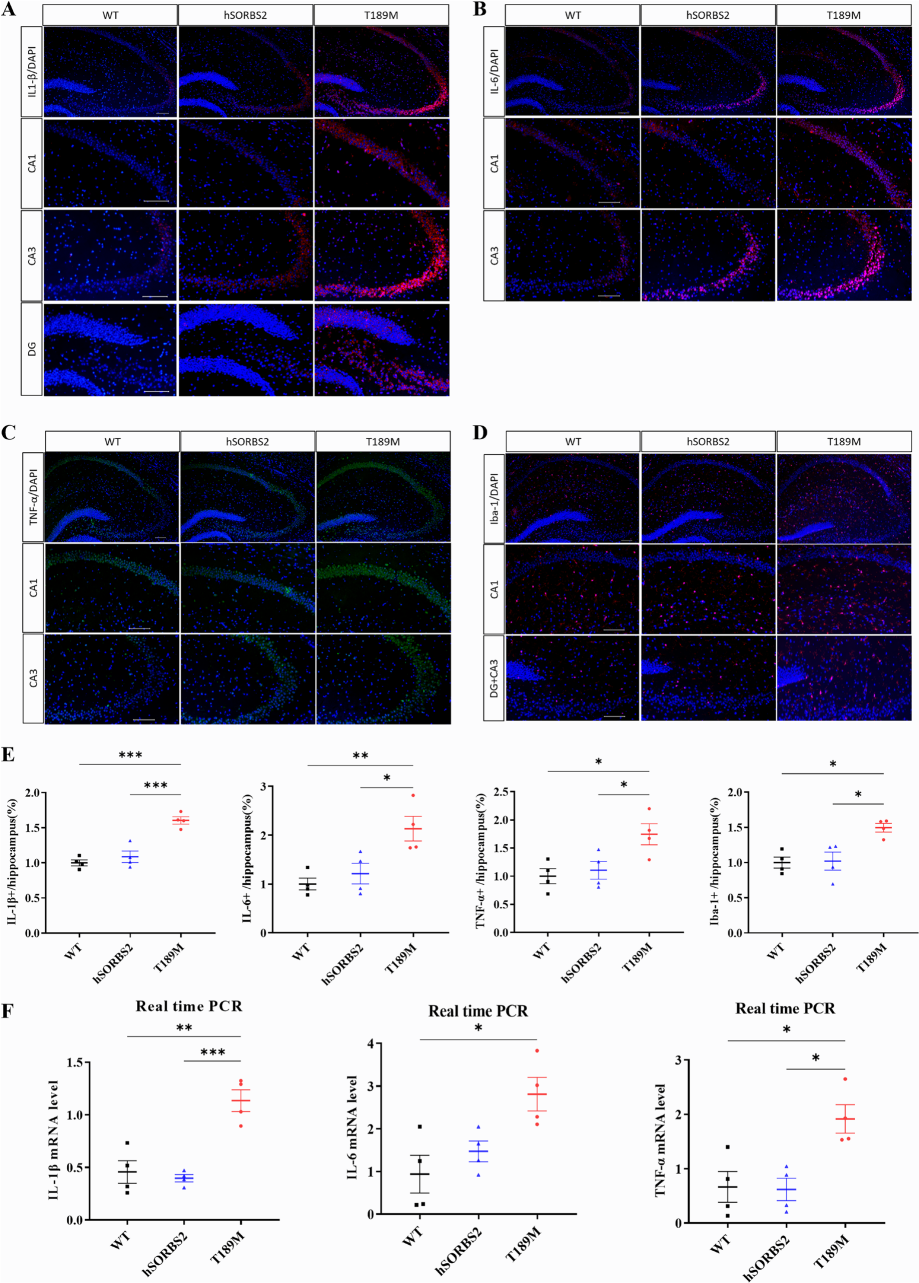
Inflammatory markers and microglia activation in the hippocampal tissues of *SORBS2* T189M transgenic mice. (A–D) Immunofluorescence of IL‐1β, IL‐6, TNF‐α, and Iba1 in the CA1, CA3, and dentate gyrus (DG) regions. (E) Quantification of IL‐1β, IL‐6, TNF‐α, and Iba1‐positive cells in the hippocampus. (F) qRT‐PCR analysis of inflammatory factor expression levels in the three groups. Scale bar, 100 μm as indicated. *n* = 4 mice/genotype; error bars SEM; one‐way ANOVA; *p < 0.05; **p < 0.01; ***p < 0.001. hSORBS2, Human SORBS2 wild‐type overexpression; T189M, SORBS2 T189M mutation; WT, wild type.

To explore the impact of the *SORBS2* T189M mutation on neuroinflammation, immunofluorescence staining and qRT‐PCR were performed. Immunofluorescence revealed increased expression of proinflammatory cytokines IL‐1β, L‐6, and TNF‐α in the hippocampus of T189M mice compared to WT and hSORBS2 mice (Figure 4A–C,E). Additionally, an increase in Iba1‐positive microglia was observed in T189M mice, which is indicative of microglial activation (Figure 4D,E). qRT‐PCR further confirmed elevated mRNA levels of IL‐1β, IL‐6, and TNF‐α in T189M mice (Figure 4F). These data suggest that the *SORBS2* T189M mutation induces neuroinflammation via microglial activation and cytokine upregulation.

### 3.6 *SORBS2* T189M Mutation Induces Neuronal Loss

**FIGURE 5:**
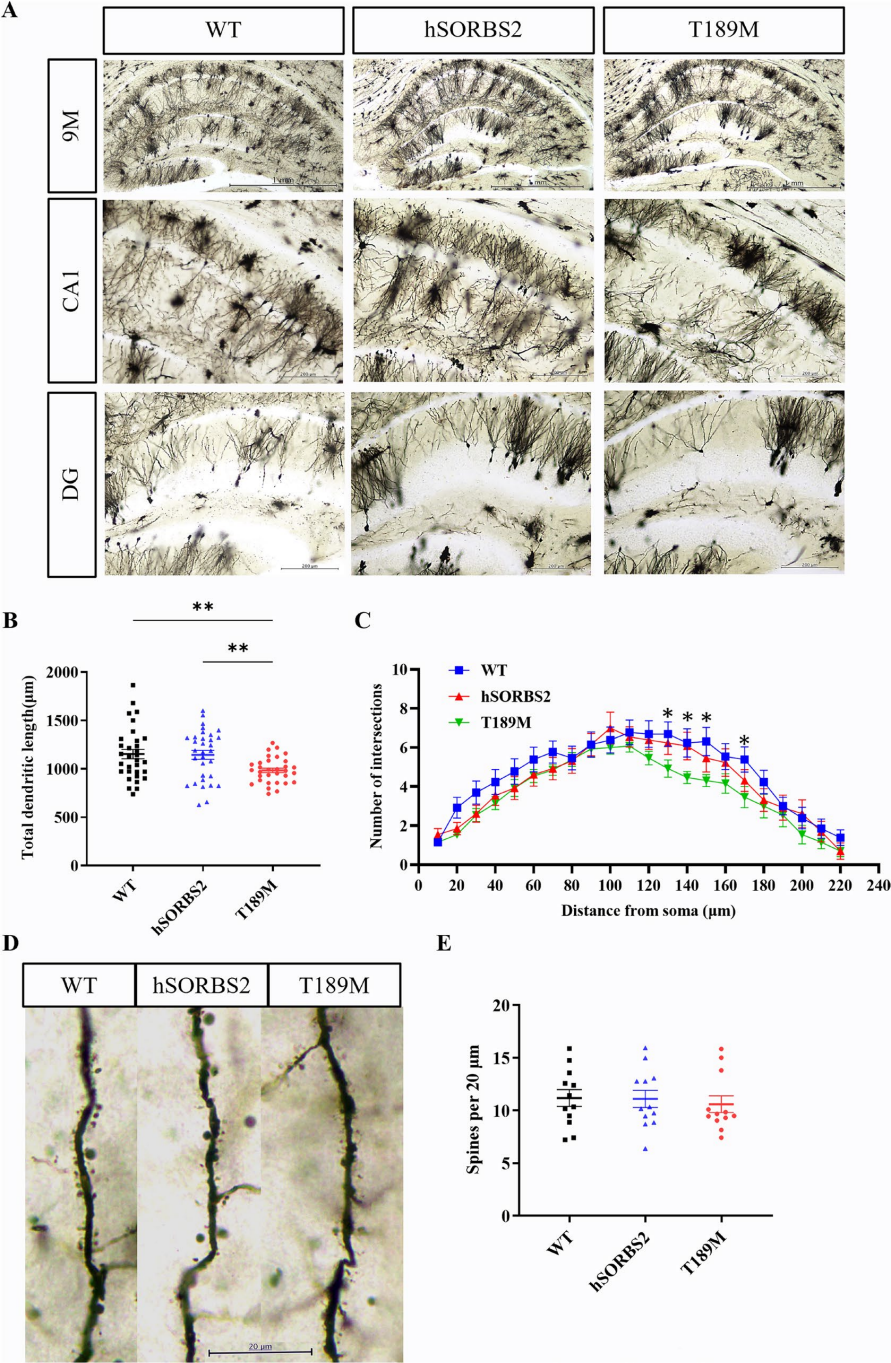
*SORBS*2 T189 mutation caused neuronal loss in transgenic mice. (A) Representative Golgi‐stained images of hippocampal sections from 9‐month‐old WT, hSORBS2, and T189M mice. (B) Quantification of total dendritic length from the soma across the three groups. (C) Quantification of dendritic branching as a function of distance from the soma. (D) Representative images of Golgi‐stained hippocampal pyramidal neurons. (E) Quantification of dendritic spine density in the images shown in D. Scale bars, 1 mm (A: Above), 200 μm (A: Below), 20 μm (D). *n* = 4 mice/genotype; error bars SEM; 8 somas from 4 mice per group (B); 3 somas from 4 mice per group (E); one‐way ANOVA; two‐way ANOVA; *p < 0.05; **p < 0.01; ***p < 0.001. hSORBS2, Human SORBS2 wild‐type overexpression; T189M, SORBS2 T189M mutation; WT, wild type.

Golgi‐Cox staining was employed to assess the impact of the *SORBS2* T189M mutation on neuronal morphology. A significant reduction of total dendritic length and the number of dendritic branches from the soma were observed in T189M mice compared to WT and hSORBS2 (Figure 5A–C). However, no significant differences were observed in dendritic spine density among the groups (Figure 5D,E). These findings indicate that the hippocampal neuronal impairment contributes to cognitive deficits observed in T189M mice.

## 4 Discussion

*SORBS2* gene is located on the 4q35. 1 which encodes the protein belongs to the adaptor protein family, and has a variety of biological effects [30, 31]. Recently, Jia et al. found that silencing *SORBS2* would aggravate free fatty acid‐induced fat accumulation and enhance inflammatory release, suggesting that SORBS2 may be a critical factor in inflammatory dysfunction [32]. Chang et al. [33] have found that reducing the expression of SORBS2 impaired the formation and morphology of dendritic spines. Feng et al. [34] demonstrated that deletion of *SORBS2* in mice may exhibit reduced dendritic complexity and impaired long‐term memory. All these suggest that *SORBS2* gene may be involved in neurological related diseases.

This study explored a four‐generation pedigree with eight members diagnosed with AD or aMCI. The clinical phenotype within this pedigree prominently involved progressive memory loss, executive dysfunction, and personality changes. ^11^C‐PIB PET imaging confirmed extensive Aβ deposition across the bilateral frontal, parietal, occipital, and lateral temporal cortices, with maximal accumulation in the right temporo‐occipital region. Whole exome sequencing revealed a novel heterozygous missense mutation, *SORBS2* T189M, which exhibited co‐segregation with the disease phenotype. Located in exon 9 of *SORBS2*, this variant has not been previously reported in AD or frontotemporal dementia (FTD) mutation databases. In silico analysis utilizing 13 predictive tools identified the *SORBS2* T189M variant as “deleterious” in 11 of them. The pathogenicity of this mutation was corroborated through transgenic mouse models, which displayed exacerbated neuroinflammation, increased Aβ accumulation, synaptic dysfunction, neuronal loss, and impaired cognitive performance (Figure 2), identifying *SORBS2* T189M as a putative pathogenic variant. To our knowledge, this is the first study implicating *SORBS2* mutations in FAD.

Our findings further demonstrated significant upregulation of IL‐1β, IL‐6, TNF‐α, and Iba1‐positive microglia in the hippocampus of T189M mice compared to WT and hSORBS2 mice, consistent with the neuroinflammatory processes characteristic of early AD. These data suggest that the *SORBS2* mutation triggers microglial activation and the production of proinflammatory cytokines, thereby contributing to the neuroinflammatory cascade. The extracellular deposition of Aβ and intracellular neurofibrillary tangle formation are well‐established neuropathological hallmarks of AD, with neuroinflammation now recognized as a core pathological feature [34]. Early microglial activation in AD instigates the release of proinflammatory cytokines, which propagate chronic neuroinflammation and accelerate disease progression [35]. IL‐1β, IL‐6, and TNF‐α are key proinflammatory cytokines central to AD pathogenesis [36], with their upregulation documented in the brains of patients with AD and transgenic mouse models recapitulating AD pathology [37, 38].

Notably, we observed a significant increase in Aβ42 levels and the Aβ42/Aβ40 ratio in the cortex of T189M mice. Pathologically, *APP* is cleaved by β‐secretase and γ‐secretase to generate Aβ40 and Aβ42 peptides [39], with Aβ42 being particularly aggregation‐prone and strongly implicated in amyloid plaque formation and neurodegeneration in AD. While mutations in *PSEN1/2* and *APP* are known to promote Aβ aggregation in FAD [40, 41], our study identifies *SORBS2* T189M as a novel pathogenic variant within a Chinese FAD family, promoting Aβ accumulation via a neuroinflammatory mechanism distinct from previously characterized FAD pathways. We hypothesized that the microglial activation and upregulated proinflammatory cytokine expression observed in this study likely contribute to the intracerebral Aβ accumulation. Overactivated microglia release large amounts of proinflammatory cytokines, chemokines, and neurotoxins, which downregulate genes involved in Aβ phagocytosis and degradation, thereby reducing Aβ clearance and promoting its accumulation. For instance, IL‐1 has been shown to upregulate *APP* mRNA synthesis and translation, implicating it in Aβ production and deposition. Elevated IL‐1β levels have also been shown to impair microglial Aβ clearance and increase blood–brain barrier permeability, exacerbating Aβ accumulation [42]. IL‐6 promotes *APP* processing and Aβ generation in primary cultured rat cortical neurons [43], while TNF‐α has been shown to increase β‐secretase (BACE1) activity, with the TNFR1 signaling pathway necessary for Aβ production in vivo [44].

Furthermore, we observed intracellular Aβ accumulation within neurons, with a lack of extracellular amyloid plaques. This observation is consistent with other AD mouse models where mutations in *PSEN* or *MAPT* genes or specific *APP* mutations, such as *APP* E693Δ‐Tg, *APP* Dutch, and *APP* Sw‐NSE, do not result in amyloid plaque formation [45, 46, 47]. Moreover, prior studies have demonstrated that neuroinflammation precedes substantial extracellular Aβ plaque deposition, with intracellular Aβ accumulation accompanied by microgliosis and increased TNF‐α levels [48, 49], consistent with our findings.

T189M mice also exhibited hippocampal neuronal damage and deficits in spatial learning and memory, likely attributable to the proinflammatory milieu within the brain. Clinical evidence suggests that microglial activation and subsequent release of proinflammatory mediators play important roles in neuronal injury and are closely associated with cognitive decline. Our data support this, indicating that microglia activation and the upregulation of proinflammatory cytokines, such as IL‐1β, IL‐6, and TNF‐α, are intimately linked with neuronal damage and cognitive dysfunction. Activated microglia can directly induce neurotoxicity through cytotoxic mediator release or indirectly via proinflammatory cytokine and chemokine production, ultimately impairing Aβ clearance and contributing to cognitive decline. Proinflammatory cytokines can also induce synaptic damage, neuronal death, and inhibition of neurogenesis through various mechanisms. For example, IL‐1β induces synaptic loss by increasing prostaglandin E2 production, leading to presynaptic glutamate release and postsynaptic N‐methyl‐D‐aspartate (NMDA) receptor activation. TNF‐α can induce neuronal death via the TNFR1/caspase‐8 pathway when the nuclear factor‐κB (NF‐κB) pathway is inhibited [35], and IL‐6 can perpetuate chronic neuroinflammation by inducing microglial release of a cascade of proinflammatory cytokines [36].

This study has some limitations. Firstly, the mouse model utilized in this study may not fully replicate human AD pathology due to the absence of extracellular Aβ plaques, a limitation inherent to all current animal models of AD. Despite the development of several transgenic mouse models harboring AD‐related mutations in *APP* and *PSEN1/2* genes, none have fully recapitulated the human disease phenotype [50]. Secondly, the specific molecular pathways and mechanisms by which *SORBS2* T189M contributes to AD pathogenesis require more in‐depth investigation. Lastly, although the *SORBS2* T189M mutation has been identified in a Chinese FAD pedigree, its relevance to AD morbidity in the population should be further studied in the future.

## 5 Conclusions

This study provides compelling evidence that the *SORBS2* T189M mutation contributes to AD pathology through mechanisms that include enhanced neuroinflammation, synaptic dysfunction, exacerbated Aβ accumulation, and neuronal loss. This mutation triggers microglial activation, leading to the excessive release of proinflammatory cytokines, which culminates in cognitive impairment in transgenic mouse models. The identification of *SORBS2* T189M as a novel pathogenic variant associated with AD underscores the pivotal role of genetic factors and neuroinflammatory processes in the disease's pathogenesis. These findings offer new insights into the molecular mechanisms underlying AD and highlight potential novel targets for therapeutic intervention.

## Author Contributions

J.J. and Q.W. designed and coordinated the study and wrote the manuscript. Q.W., S.W. and S.C. carried out most studies and data analysis. J.J. found the FAD pedigree. Q.W. conducted the FAD pedigree follow‐up. Y. Wei managed mouse lines and conducted the Morris water maze test. Y.L. conducted immunohistochemical experiments. Y. Wang, Y.L., W.Q. and M.Q. conducted the FAD pedigree sample collection, neuropsychological and neuroimaging assessment. J.J. supervised the project. All authors read and approved the final manuscript.

## Ethics Statement

This study was approved by the Ethics Committee of Xuanwu Hospital, Capital Medical University (Approval No.: LYS [2019]110). Samples were collected in accordance with the principles of the Declaration of Helsinki. All animal procedures used for this study were prospectively reviewed and approved by the Ethics Committee of Capital Medical University (Approval No.: AEEI‐2017‐004).

## Consent

Informed consent was obtained from all participants or their legal representatives.

## Conflicts of Interest

The authors declare no conflicts interests.

## Supporting information


Data S1.

## Data Availability

The datasets supporting the conclusions of this article are included in the article.
