# Supplementary material for: A Novel Missense Variant in SORBS2 Is Causative With Familial Alzheimer's Disease
Source: CNS Neurosci Ther. 2025 Feb 6;31(2):e70256. doi: 10.1111/cns.70256 (PMC11800137; doi:10.1111/cns.70256)
Supplement: Supplementary file 1 — Data S1. [file CNS-31-e70256-s001.docx]

**Supplementary materials**

**Supplementary Table 1 Primers used for qRT-PCR analysis**

| Gene | Forward (5′–3′) | Reverse (5′–3′) |
| --- | --- | --- |
| *SORBS2* | CAGGGCGTGATTCTCAGTCA | AGGTTTGGTCGAACGCTTCT |
| *TNF-α* | GTGGTCAGGTTGCCTCTGTCTC | TGGCTCTGTGAGGAAGGCTGTG |
| *IL-1β* | TTTCCTCCTTGCCTCTGATGGG | CCACACGTTGACAGCTAGGTTC |
| *IL-6* | CTTGGGACTGATGCTGGTGACA | GCCTCCGACTTGTGAAGTGGTA |
| *GAPDH* | GAAGGGCATCTTGGGCTACAC | GTTGTCATTGAGAGCAATGCCA |

**Supplementary Table 2 Pathogenicity predictions for identified segregating variants**

| **GeneSymbel** | ***WDR65/CFAP57*** | ***SORBS2*** | ***ZFR*** | ***ITGA2*** | ***GARS*** | ***PLA2G4F*** | ***UBR1*** |
| --- | --- | --- | --- | --- | --- | --- | --- |
| **Transcript ID** | NM_001195831.2 | NM_001270771.1 | NM_016107.3 | NM_002203.3 | NM_002047.2 | NM_213600.3 | NM_174916.2 |
| **cHGVS** | c. 3350G>A | c. 566C>T | c. 1100A>G | c. 2269C>T | c. 2212G>A | c. 1459C>T | c. 2480C>T |
| **pHGVS** | p. R1117Q | p. T189M | p. N367S | p. R757C | p. E738K | p. R487C | p. S827L |
| **SIFT4G_pred** | T | D | T | T | T | T | T |
| **Polyphen2_HDIV_pred** | NA | D | B | NA | P | D | B |
| **Polyphen2_HDIV_score** | NA | 1.000 | 0.001 | NA | 0.760 | 0.975 | 0.085 |
| **Polyphen2_HVAR_pred** | NA | D | B | NA | B | P | B |
| **Polyphen2_HVAR_score** | NA | 0.999 | 0.001 | NA | 0.091 | 0.498 | 0.026 |
| **Fathmm_pred** | T | T | T | T | D | T | T |
| **Fathmm-MKL_coding_pred** |  |  |  |  |  |  |  |
|  | D | D | D | D | D | T | D |
| **Fathmm-XF_coding_pred** | T | D | T | T | D | T | T |
| **LRT_pred** | N | D | D | N | NA | N | D |
| **MutationTaster_pred** | N | D | D | D | D | N | D |
| **PROVEAN_pred** | N | D | N | N | N | NA | N |
| **MetaLR_pred** | T | T | T | T | T | T | T |
| **GERP++_RS** | 4.030 | 4.860 | 4.390 | 0.896 | 4.900 | -0.372 | 3.940 |
| **LoFtool** | 0.953 | 0.717 | 0.098 | 0.501 | 0.290 | 0.813 | 0.598 |
| **Condel** | N | D | N | N | N | D | N |
| **CADD_phred** | 22.5 | 28.1 | 21.3 | 22.8 | 27.9 | 22.2 | 23 |
| **CADD_raw** | 2.409 | 4.689 | 2.046 | 1.872 | 3.622 | 2.155 | 2.531 |
| **DANN_score** | 0.994 | 0.999 | 0.931 | 0.899 | 0.998 | 0.987 | 0.383 |
| **M-CAP_pred** | T | D | T | T | D | T | T |
| **M-CAP_score** | 0.004 | 0.054 | 0.005 | 0.016 | 0.061 | 0.004 | 0.007 |
| **Total damaging variant score (0-20)** | 5 | 17 | 7 | 3 | 11 | 6 | 5 |

NA: not available. **SIFT4G_pred** D: deleterious (sift≤0.05); T: tolerated (sift>0.05). **Polyphen2_HDIV_pred** D: Probably damaging (>=0.957); P: possibly damaging (0.453≤pp2_hdiv≤0.956); B: benign (pp2_hdiv≤0.452). **Polyphen2_HDIV_score** Higher scores indicate a greater likelihood of structural or functional changes in the protein. **Polyphen2_HVAR_pred** D: probably damaging (≥0.909); P: possibly damaging (0.447≤pp2_hvar≤0.909); B: benign (pp2_hvar≤0.446). **Polyphen2_HVAR_score** Higher values suggest a greater probability of deleterious effects. **Fathmm_pred** D: deleterious; T: tolerated; lower values indicate higher deleterious potential. Fathmm predicts the functional consequences of both coding and noncoding variants in the human genome. **Fathmm-MKL_coding_pred** D: deleterious; T: tolerable. **Fathmm-XF_coding_pred** D: deleterious; T: tolerated. This model provides accurate predictions of pathogenic point mutations using extended features. **LRT_pred** D, deleterious; N, neutral; U, unknown. Higher values indicating a greater likelihood of structural or functional changes. **MutationTaster_pred** A: disease-causing automatic; D: disease-causing; N: polymorphism (probably harmless); P: polymorphism automatic (known to be harmless); higher scores denote greater deleteriousness. **PROVEAN_pred** D: deleterious; N: neutral; higher scores denote greater deleteriousness. **MetaLR_pred** D: deleterious; T: tolerated; higher scores indicate a greater probability of deleterious effects. **GERP++_RS** GREP++ "rejected substitutions" (RS) score; higher values suggest greater deleterious potential. **LoFtool** P: probably damaging; D: damaging; B: benign; LoFtool ranks genetic intolerance based on the loss-of-function ratio, with lower gene score percentiles, indicating greater intolerance to functional variation. **Condel** Condel combines high-throughput sequencing data to identify copy number variations and deletion types. D: deleterious; N: neutral. **CADD_phred** CADD Phred-like scores; higher values indicate a greater probability of deleterious effects. **CADD_raw** CADD raw scores: Higher values suggest higher deleterious potential. **DANN_score** deleterious annotation of genetic variants using neural networks; higher values are considered more deleterious. **M-CAP_pred** D: deleterious (M-CAP score >0.025); T: tolerated. **M-CAP_score** M-CAP predicts the pathogenicity of rare missense variants. Variants lacking an M-CAP score should be presumed likely benign.

**Supplementary Figure 1**


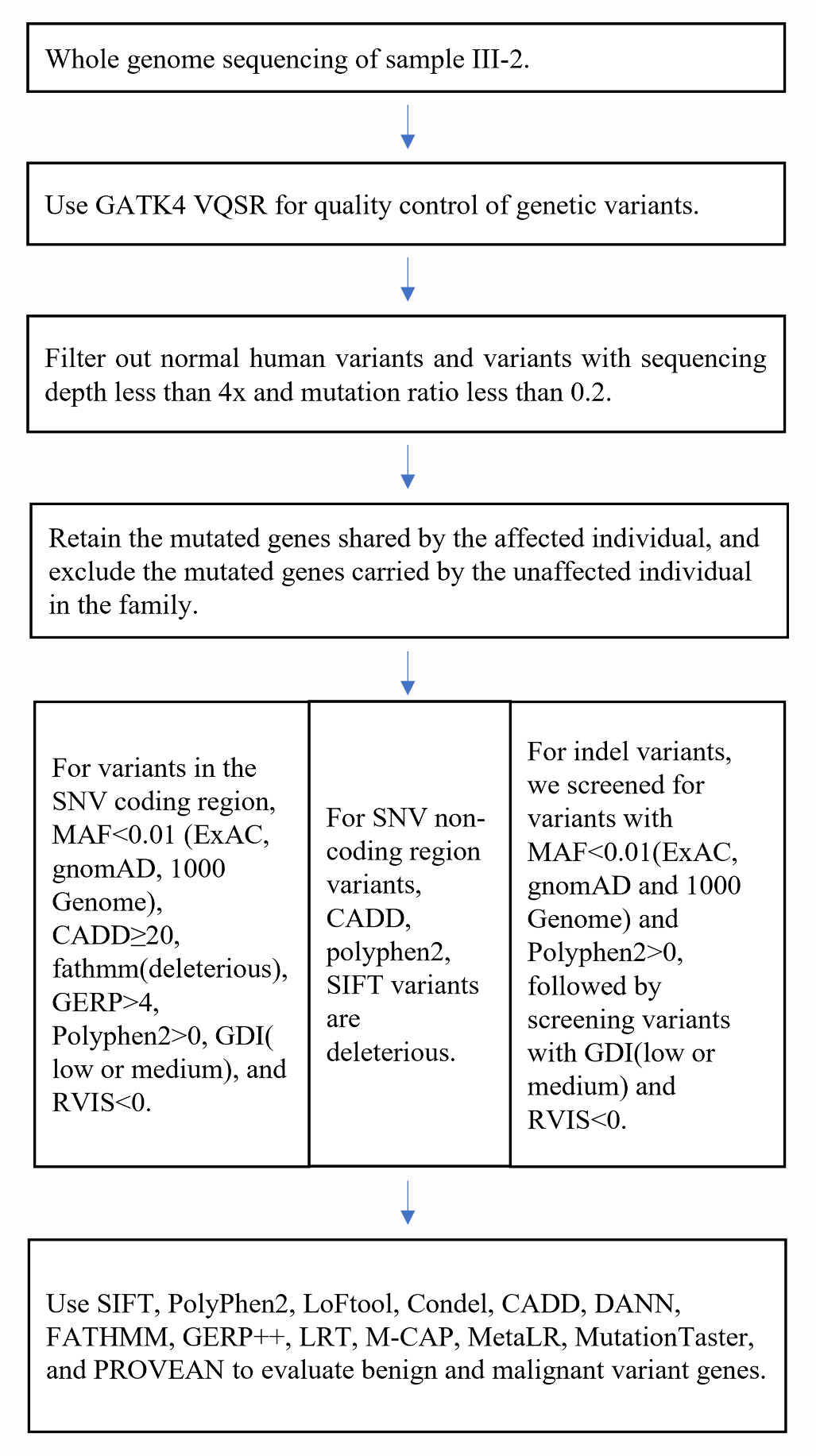


**Supplementary Fig 1 Schematic representation of the identification process for candidate genetic variants.**
